# Axolotl mandible regeneration following complete transverse amputation involves a blastema formation and exhibits a limit along the proximodistal axis

**DOI:** 10.1371/journal.pone.0348286

**Published:** 2026-05-21

**Authors:** Samanta Tarquino González, Belfran Carbonell-Medina

**Affiliations:** 1 Departamento de Estudios Básicos Integrados, Facultad de Odontología, Universidad de Antioquia, Medellín, Colombia; 2 Grupo Genética, Regeneración y Cáncer, Facultad de Ciencias Exactas y Naturales, Instituto de Biología, Universidad de Antioquia, Medellín, Colombia; Tokai University, School of Medicine, JAPAN

## Abstract

Current therapeutic strategies aimed at promoting the regeneration of craniofacial tissues do not fully address all physiological and aesthetic needs. This highlights the need to investigate the regenerative response of craniofacial tissues in regenerative species like *Ambystoma mexicanum* (*A. mexicanum*), particularly concerning the mandible. However, although previous studies have demonstrated the regenerative response after lateral cuts of the mandible in *A. mexicanum*, it is unknown whether it can regenerate the mandible after complete transverse amputation, a type of injury used to remove tumors and extensive lesions that involve the symphyseal and parasymphyseal. Furthermore, it remains uncertain whether there is a limit to the regenerative response along the mandible proximal-distal axis. In light of this, we assessed the regenerative response following axolotl mandible complete transverse amputation through macroscopic and histological analyses and skeletogenic assessment via diaphanization. We also evaluated proliferation and the expression of genes associated with blastema formation. The results indicate that following mandibular injury, a blastema formed from which all amputated tissues were regenerated by 180 days post-amputation (dpa) and integrated seamlessly with the remaining structures. However, morphological alterations were noted in the regenerated skeletal elements. Additionally, we identified the expression of genes characteristic of epimorphic regeneration, including *Prrx1, Kazald1, Msx2, Pax7*, and *Sox2*. Interestingly, regenerative failure was observed after a second, more proximal amputation. Together, our findings suggest that the regenerative response to complete transverse amputation of the mandible in axolotls is fundamentally epimorphic; however, this regenerative capacity appears to have limits along the mandible proximal-distal axis.

## Introduction

Regeneration biology has focused on understanding tissue regeneration in vertebrate species with high regenerative capacities, including zebrafish and urodele amphibians such as *A. mexicanum* [1–4]. The latter is a salamander species capable of regenerating different structures such as limbs, tail, heart, dermis, iris, and, of great interest, craniofacial structures [5–11]. Additionally, *A. mexicamum* possesses similarities to vertebrates concerning developmental processes, anatomy, and morphology of craniofacial structures such as the mandible [12–17]. The above places this species as an excellent model to study the mechanisms underlying the regenerative response of craniofacial tissues [6,12].

The regeneration of craniofacial tissues has been a topic of great interest given the need to develop new therapies to improve the quality of life of those who have lost some craniofacial structure or tissue [18,19], considering that damage to these structures leads to a notable deformity affecting the aesthetics and functions of the craniofacial system [18,20]. The mandible represents one of the regions of the craniofacial complex most exposed to trauma and affectations by pathologies such as cancer, which, depending on the tissues involved and the extent of the affected tissue, the patient can be intervened for the removal of medium or large extensions of mandibular tissue to finally undergo reconstructive surgeries, where the endogenous reparative response of the remaining tissues is fundamental for the success of the treatment [12,18,21–23]. However, even though current treatments such as autografts, allografts, alloplastic prostheses and scaffolds have offered favorable results, these treatments have limitations and complications such as low availability of the required tissue, scarring problems, rejection or atrophy of the graft due to lack of integration of the transplanted tissue, donor site morbidity and, in general, low regenerative response of the intervened site [18,23–28]. Accordingly, previous work has designed different models of mandibular injury in species with the capacity to regenerate the mandible to simulate the surgical approaches performed in humans to evaluate the regenerative capacity of the remaining tissues after mandibular amputation. Therefore, the ultimate goal is to understand the cellular and molecular mechanisms involved in the regenerative response of this amputated structure. Thus, models of unilateral transverse and complete transverse parasymphyseal injury have been developed in different adult salamanders and newts *(Ambystoma maculatum, Triturus Viridescens, Cynops pyrrhogaster)* [29–33], as well as in the teleost *Danio rerio* [34–36]. These studies show that the regenerative response of the mandible in these species occurred by an epimorphic regeneration mechanism; however, the regenerated tissues were not morphologically identical to those initially amputated. Regarding *A. mexicanum*, previous studies have evaluated the regenerative response in punch-type soft tissue injury models on the floor of the mouth [6], dentectomy with subtle removal of bone tissue [5], and recently, lateral cuts in the mandible [7]. However, to date, the regenerative response of the mandible following a complete cross-section involving the region of the two hemimandibles in a maximum defect has not been evaluated. Additionally, it is unknown whether this regenerative response is mediated by blastema formation, which is characteristic of epimorphic regeneration in this type of lesion [37,38]. Therefore, to address this lack of knowledge, this study aimed to design a surgical strategy involving both adult *A. mexicanum* hemimandibles and to evaluate the regenerative response from macroscopic, histological, skeletogenic, and gene expression described during the epimorphic regenerative response. The results of this study show that the regenerative response after complete mandibular transverse amputation involved the formation of a blastema from which the amputated tissues were regenerated and integrated with the remaining tissues; however, the regenerated tissues presented morphological alterations. On the other hand, this regenerative response failed when a more proximal amputation was performed along the mandibular axis. Overall, the results of this study raise new questions about the mechanisms and signalling pathways that regulate the regenerative response following mandibular amputation, as well as the development of new studies to identify what factors are involved in the failure of the regenerative response when the mandible is amputated proximally and how this may relate to the inability of other species such as mammals and humans to regenerate the mandible and other structures.

## Materials and methods

### Animal and ethical treatment

Adult animals of the *A. mexicanum* species (axolotls) of wild phenotype (15 cm snout to tail) were used. The animals stayed in the Genetics, Regeneration and Cancer laboratory of the SIU (Sede de Investigación Universitaria) at the Universidad of Antioquia-Colombia, under the same conditions, fed with FLYMEAT protein pellets (A Lot’l Axolotls, Ontario Canada) for salamanders and temperature between 19 and 21°C in 20% Holfreter’s solution. These animals were from the Ambystoma Genetic Stock Center *(AGSC)* at the University of Kentucky. The animal experimentation procedures were approved by the Ethics and Animal Experimentation Committee of the University of Antioquia under Act No. 151 and the Ethics Committee of the Faculty of Dentistry of the University of Antioquia in Act 03 of 2023.

### Surgery and regeneration assays

The animals were anesthetized with Tricaine Methanesulfonate (MS-222) 0.1% (Sigma, St. Louis, Missouri) for 15 minutes for all surgical and tissue collection procedures, according to [39,40]. A complete transverse cut was made at the level of the distal mandibular third, involving both hemimandibles (removal of the entire symphyseal region) and amputating approximately 2.5 mm in the chin region, based on the technique used by Goss and Stagg in the *N. Viridescens* model [31]. Subsequently, 0.5% Sulfamerazine (Sigma, St. Louis, MO) was applied, and the animals were kept in 40% Holtfreter solution for future observations. A second, more proximal amputation was performed 2 mm away from the first amputation for histological, gene expression, and regenerative response monitoring analyses.

### Macroscopic, histological and skeletal characterization

**Fig 1:**
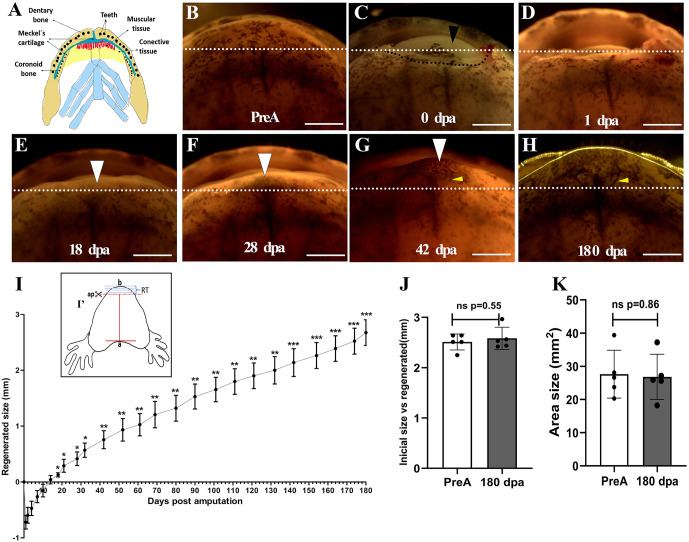
Macroscopic characterization of the mandibular regeneration process following complete transverse amputation. **(A)**, Representative schematic of the structures forming the mandible in A. mexicanum. Illustration created by the authors based on original photographs taken by the authors. The photographs were used solely as anatomical references and are not reproduced in the manuscript. **(B)**, Pre-amputation. **(C)**, Post-amputation (0 dpa), amputation of approximately 2.5 mm of tissue, black arrowhead points to the tongue. A black dotted line indicates the retraction of the remaining soft tissue. **(D)**, 1 dpa, wound closure. **(E)**, 18 dpa, formation of an ‘early blastema’ (white arrowhead). **(F)**, 28 dpa, ‘mid-pigmented blastema’ (arrowhead). **(G)**, 42 dpa, ‘late blastema’. Yellow arrowhead indicates neovascularization. **(H)**, 180 dpa, total regeneration. **(I)**, Regenerative tissue growth curve. (**I‘**), Schematic represents how regenerative tissue length (RT) was determined. ap: amputation plane. Illustration created by the authors based on original photographs taken by the authors. **(J)**, Quantification of pre-amputation size versus regenerated size. **(K)**, Quantification of measured regenerated area. This measurement corresponds to the area between the amputation plane and point “b”. **(L)**, Quantification of regenerated area measured in mm^2^. Data plot mean ± SEM. *:p < 0.05; **: p < 0.01; ***: p < 0.001. PreA: Pre-amputation, dpa: days post-amputation. A White dotted line indicates the amputation plane. Scale bar = 2 mm. An n = 6 animals were used for these analyses.

Following the surgical treatments, photographs were captured using a stereomicroscope (Olympus SZX16, Tokyo, Japan) in conjunction with a digital camera (MotiCAM 5, Kowloon, Hong Kong) and analyzed with Motic Images Plus software (Version 2.0) to document the follow-up and macroscopic assessment of the regenerative response from 24 hours post-amputation (hpa) to 180 days post-amputation (dpa). An *n = 6* animals were used for macroscopic analysis. Measurements, including the total regenerated area and the proximal-distal length of the regenerated tissue, were taken and compared with those of the pre-amputation tissue. The proximal-distal length of the regenerated tissue (RT) was equal to the subtraction of the longitudinal measurement obtained from a fixed point ‘a’ (mandibular fold) to the most distal point of the regenerating tissue **‘b’** minus the distance from the mandibular fold **´a´** to the amputation plane line **‘ap’** at 0 dpa (Fig 1I). For histological analysis, tissues were collected at 0 dpa, 1 dpa, 18 dpa, 28 dpa, 42 dpa and 180 dpa (*n = 3 – n = 6 animals for each observation time*). The tissues were fixed in 4% PFA (paraformaldehyde, Sigma Saint Louis, MO 63103, USA), embedded in paraffin, and cut into 3 µm sections. The samples were then processed with Masson’s Trichrome staining [41]. Additionally, animals that underwent a second proximal amputation for tissue collection at 18 dpa, 28 dpa, 42 dpa and 180 dpa underwent macroscopic follow-up until 180 days post-reamputation (dpr) as well as histological and skeletal analysis (*n = 3 animals exposed to a second amputation*). Similarly, other animals were kept under observation for up to 18 months after the second proximal amputation (approximately 545 days).

For regenerated skeletal tissue characterization, animals were euthanized according to [42] and diaphanization technique was performed with alcian blue and alizarin red staining at 180 dpa and 180 dpr (*n = 4*) according to the protocol described previously [39,43]. Samples were fixed in 4% PFA for 48 hours and washed with distilled water every 8 hours for 2 days. Carefully, skin and muscle tissue were removed, and the samples were deposited in 0.3% alcian blue solution for 48 hours at 37°C; rehydrated in ethanol series washes for 2 hours, and washed with distilled water for 2 hours. The samples were treated with 1% trypsin/saturated sodium borate solution for 24 hours and immersed in 0.5% alizarin red/KOH solution for 24 hours. Finally, five washes were performed with 0.5% KOH each two hours and were immersed in KOH1%/Glycerol solution of 3:1, 1:1 and 1:3 series for 24 hours each one, leaving the samples in 87% glycerol for photography and analysis by stereomicroscope (Olympus SZX16, Tokyo, Japan) and digital camera (MotiCAM 5, Kowloon, Hong Kong).

### RNA extraction and RT-qPCR

Total RNA extraction was performed from non-regenerating mandibular tissue (pre-amputation) and regenerating tissues at 18 dpa and 28 dpa by the Trizol method (Thermo Fisher Scientific, Carlsbad, CA, USA; catalog number 15596026) following the manufacturer’s protocol. Subsequently, cDNA was synthesized using RevertAid H Minus Strand cDNA synthesis kit (Thermo Scientific, Vilnius, Lithuania; catalog number K1632) from 500 ng of total RNA, pretreated with RNase-free DNase I. The cDNA was diluted 1:10 before qPCR assays. The q-PCR reactions were performed using the iQ SYBR Green mix and an iQTm iCycler iQTm detection system (Bio-Rad Laboratories, Hercules, CA, USA; catalog number 1708880). Gene expression levels were normalized to endogenous *18S* reference gene expression as previously reported [44]. Expression of *Prrx1, Kazald1, Msx2, Klf4, Nanog, Sox2, Col2A1, Myf5,* and *Pax7* genes was evaluated in three independent biological replicates with their respective technical triplicates for each gene and day to be evaluated. The sequences of primers used are listed in Table 1. In addition, negative controls without the first cDNA strand were performed for each gene evaluated. Expression levels were quantified using the 2^-ΔΔΔ^ CT method [49].

**Table 1:** List of primers used for qPCR.

Gene	Sequence	Amplified product size	Reference
*Prrx-1- F*	CCCAGACGCCTTTGTAAGAG	98	[45]
*Prrx-1- R*	GGCGAAACTTTGCTCTTCGG		
*Msx2-F*	TCCATTGCTGAGAGAGCAGA	232	[45]
*Msx2-R*	TTGGTCTGTGGAAGGGGTAG		
*Sox2-F*	CTGCAGTACAACTCCATGAATCC	239	[46]
*Sox2-R*	ATGCTAATCATGTCCCTCAGGTC		
*Nanog-F*	ACTTTACCAAAAAGCGTGACACTAGA	79	[47]
*Nanog-R*	ACAGAGCACCCAATTTTCCAA		
*Myf5 -F*	GGGAGCCCCCTTTCCAA	87	[47]
*Myf5 -R*	GGCGCTGTCAAAGCTGTTG		
*Pax7 -F*	AAACCAAGCACAGCATCGAC	99	SalSite: contig606468
*Pax7 -R*	TGCGTTTCAAGGGCAAGTC		
*Col2A1-F*	CCTATGGACATTGGTGGTGC	303	SalSite: FT9N62301B1T8S
*Col2A1-R*	GGAGGGAAACAAAGCAAGCA		
*Kazald1-F*	GAAAATGGATAAGGTGGTGGGGAGGG	112	[48]
*Kazald1-R*	CTCGTGACATCCTGAGCCTGGAAG		
*Klf4-F*	GCTCTTGGTGTAGGTCTTGC	202	SalSite: Contig144932
*Klf4-R*	CCGCCCTCTTCACCTTTAGA		

### BrdU and immunofluorescence assay

Intraperitoneal injection of BrdU (5-Bromo-2-deoxyuridine) (Sigma, United States) (0.25 mg/ g animal weight) was performed in two pulses of 24 and 48 hours prior to regenerative tissue collection and pre-amputation. Pre-amputation tissue of interest was collected at 0 dpa,1 dpa,18 dpa, 28 dpa and 42 dpa (n = 3 for each point to be evaluated) and fixed in 4% PFA and embedded in paraffin for subsequent 3 µm histological sections for subsequent immunofluorescence according to [40]. Slides were deparaffinised for 1 hr at 60 °C, rehydrated in ethanol series (100%, 95%, 85%, 75% and 50%), then fixed in 4% PFA for 5 min followed by 3 washes for 5 min with 1X TBST (1X Tris-buffered saline, 0.1% Tween 20). Treatment was performed with 2M HCl at 37°C for 30 min and neutralised with 3 washes of 1X PBS (phosphate buffered saline) pH 7.4 for 10 min. The sections were then incubated in blocking solution (10% foetal bovine serum in 1X TBST) for 3 h at room temperature and then incubated with anti-BrdU primary antibody (1:500) [50] at 4°C overnight. The next day, they were washed with 1X TBST and incubated with anti-mouse Alexa fluor 594 secondary antibody (1:200, Abcam #ab150120). Subsequently, nuclear staining was performed with Hoechst (1:1000).

Images were acquired using AXIO VISON ZOOM Carl fluorescence microscopy and processed for quantification by means of a script designed in Colab-Python software, selecting a 600 x 600 µm region of interest including bone tissue and remnant cartilage, epithelium, and blastema tissue. Between 5–6 histological sections were used for each biological replicate (*n = 3*). Proliferation was quantified as a proliferative index, calculated as the percentage of BrdU^+^ nuclei relative to the total number of Hoechst-labeled nuclei. This normalization accounts for differences in tissue size and total cell number among samples. These proliferation levels were compared to non-regenerative tissues pre-amputation.

### Statistical analysis

For data analysis, the normality and the homogeneity of variance of the dataset were assessed using the Shapiro-Wilk test and Levene’s test. Comparisons among different regeneration points concerning size and area were conducted using the paired Student’s t-test. In evaluating the regenerated skeletal tissue, the size of the regenerated structures was compared to that of the pre-amputation structures using the independent Student’s t-test. To assess cell proliferation levels, One-way ANOVA with Tukey’s post hoc test for multiple comparisons was used. For qPCR data, gene expression levels were compared between intact jaw and 18 dpa or 28 dpa samples and t-student test was applied for unpaired independent samples, and in the case of data with differences in equality of variances the adjusted Welch’s test was performed, with a significance level of p < 0.05. To account for multiple comparisons across the nine analyzed genes, p-values were adjusted using the Benjamini–Hochberg false discovery rate (FDR) method, with an adjusted p < 0.05 considered significant. A significance level of p < 0.05 was established for all statistical tests.

## Results

### Mandibular regeneration after complete transverse amputation in *A. mexicanum* involves the formation of a potential blastema

To characterize mandibular regeneration in *A. mexicanum*, a complete cross-section of the mandible (including both left and right hemimandibles) was made by amputating 2.5 mm from the amputation plane to the distal region of the mandible (*n = 6 animals*) (Fig 1B). The amputated tissue included skeletal structures such as Meckel’s cartilage and dental bone, soft tissue (muscle and skin) and dental structures (Fig 1A). Following amputation, a rapid hemostatic response was observed, accompanied by immediate retraction of the remaining soft tissue, which was evident by the concavity of the remaining tissue (Fig 1C). At 1 dpa, wound closure is observed, and retraction of the remaining soft tissue is less pronounced, with no bleeding or signs of inflammation evident (Fig 1D). At 18 dpa, there is apparent tissue growth extending from the amputation plane towards the distal region giving rise to a protuberance indicating the formation of a possible “early blastema” (Fig 1E). At 28 dpa, the size of the protrusion becomes more pronounced and the presence of black pigmentation is identified in the central and more distal region of the growing tissue, giving the appearance of a pigmented blastema “pigmented mid blastema” (Fig 1F).

Of great interest, at 42 dpa, the central pigmented area of the late blastema is similar to the skin pigmentations observed in the pre-amputated tissue, and an apparent neovascularisation of the blastema is identified (Fig 1G). Additionally, the regenerated mandibular tissue now has a more arched morphology like the shape of the pre-amputated mandibular tissue (Fig 1G). During the regeneration period from 42 dpa to 180 dpa, the regenerating structure exhibits a localized growth pattern, primarily in the more distal and medial regions, characterized by a marked pigmentation of the regenerating tissue. This is followed by uniform lateral growth towards the ends, providing continuity and shape to the mandibular arch. At 180 dpa, complete regeneration of the size and area of the amputated tissue was observed macroscopically, achieving the initial length and shape of the amputated mandible *(n = 6)* (Fig 1H). Subsequently, the size of the regenerating structure was quantified, and a growth curve was generated (Fig 1I). At the first 7 dpa, the remaining tissue retracts approximately 0.7 mm and recovers its original position until 14 dpa (Fig 1I). From day 18 until 180 dpa, the growth of the structure was statistically significant (Fig 1I). Thus, the comparison of the final regenerated size at 180 dpa with the pre-amputation size using Student’s t-test, indicated no significant difference (*p = 0.55*). This suggests that the amputated structure has regenerated to its original size (Fig 1J). However, considering the increase of regenerated tissue size in the growth curve, this growth was relatively slow. Regarding the area of the regenerated tissue, the measurement at 180 dpa was 28.07 mm² (with a SEM of 5.395), compared to the pre-amputation tissue area of 27.79 mm² (with a SEM of 4.529 mm²). Again, there were no significant differences found (*p = 0.86*), indicating that the amputated jaw area recovers pre-amputation dimensions by 180 dpa (Fig 1K).

### Histological characterization of regenerating tissues after mandible complete transverse amputation

**Fig 2:**
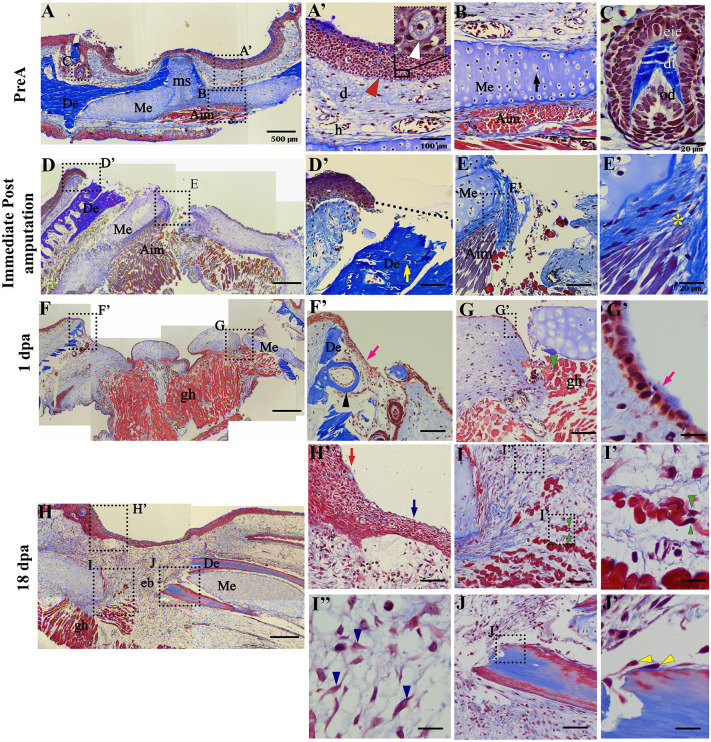
Histological analysis of the early regenerative response. **(A)**, Pre-amputation tissue, the different tissues that make up the jaw are observed. (**A’**), Magnification of the skin area; inset indicates Leydig cell, e: epithelium; d: dermis; h: hypodermis. **(B)** Magnification of the area of Meckel’s cartilage and adjacent muscle tissue. **(C)** Developing tooth germ with its component layers od: odontoblasts, dt: dentine, eie: inner enamel epithelium. **(D)** Immediate post-amputation tissue, loss of continuity of tissues is visible. (**D‘**) Magnification of remnant epithelium area followed by loss of tissue continuity and exposed wound; dotted line indicates amputation plane. **(E)** Magnification of exposed muscle and cartilage tissue. (**E’**) Magnification of interaction of the anterior intermandibular muscle (Aim) with the perichondrium at the wound edge (yellow asterisk). **(F)** Tissue 24 hpa, remnant epithelium followed by a monolayer of epithelium overlying the wound and remnant skeletal tissues. (**F’**), Zoom of remnant epithelial tissue and regenerated epithelial monolayer overlying remnant bone tissue (pink arrow) and teeth embedded in it (black arrowhead). **(G)**, Epithelial, connective, and muscle tissue underlying the amputation plane. Fragmented muscle fibers are observed (green arrowhead). (**G‘**), Epithelial monolayer covering the wound edge. **(H)**, Early blastema (eb) at 18 dpa. (**H’**), Zoom of remnant stratified epithelial tissue (red arrow) followed by regenerated epithelial tissue (blue arrow). **(I)**, Area of blastema adjacent to skeletal tissue and remnant muscle. (**I‘**) Muscle fiber in apparent fragmentation (green arrowhead). (**I’‘**) Zoom of fibroblastoid-like cells (blue arrowhead) composing the forming blastema. **(J)** Remnant bone tissue in apparent histolysis. (**J’**), Zoom of **J.** Yellow arrows: represent cells detached from the periosteum. **De**, dental bone; **gh,** geniohyoideus muscle**; Me**, Meckel’s cartilage. Scale bar in A, D, F, and H: 500 µm. Scale bar in A´, D´, F´, H´, B, E,G,I and J: 100 µm. Scale bar in C,E´, G´, I´, J´and I´´: 20 µm.

**Fig 3:**
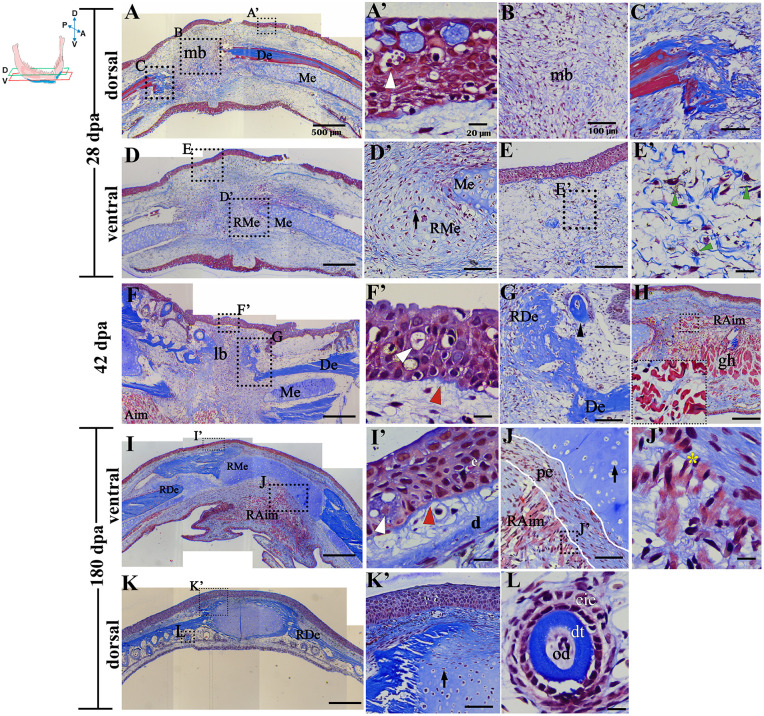
Histological analysis of the mid and late regenerative response. **(A)**, Dorsal section of regenerating tissue at 28 dpa. (**A‘**), Leydig cells and epithelium with the absence of basal lamina. **(B)**, Cumulus of cells of fibroblastoid aspect and immature extracellular matrix in the mb. **(C)**, Histolysis region of remnant bone tissue. **(D)** Ventral section of regenerating tissue at 28 dpa. (**D’**), Ventral section of regenerating tissue at 28 dpa. (**D‘**), Zoom of the chondrogenic zone. (**E**) and (**E’**), presence of pigmented cells (green arrowhead). **(F)**, Late blastema at 42 dpa. (**F’**), Stratified regenerating epithelial tissue, Leydig cells and distinguishable basal lamina. **(G)**, Zoom of regenerating bone tissue with forming teeth. **(H)** Aimrg fibres adjacent to the perichondrium of the remaining bone tissue. **(I – K)**, sections in the dorsal and ventral planes at 180 dpa. (**I’**) Zoom of regenerated epithelial tissue. **(J)**, Regenerated cartilage and muscle. **(J’)** insertion of regenerated muscle tissue in the perichondrium (yellow asterisk). **(K)**, Cut at dorsal level at 180 dpa, regenerated Meckel’s cartilage is observed at central level surrounded by dental bone and regenerated dental tissue. (**K’**), Central zone of regenerated Meckel’s cartilage and adjacent bone tissue. **(L)**, regenerated dental tissue. **AimRg**, anterior intermandibular muscle regenerated. **od**, odntoblasts. **dt**, dentin. **eie**, internal enamel epithelium. **e**, epidermis. **d**, dermis. **De**, dental bone. **gh,** geniohyoideus muscle. **mb**: medium blastema. **Me**, Meckel’s cartilage.**pe**, perichondrium. RMe, Regenerated Meckel’s cartilage. RDe, Regenerated dental bone. White arrowhead indicates Leydig cell with granular cytoplasm. Red arrowhead, indicates basal lamina. Black arrow, indicates chondrocytes. Yellow arrow, indicates osteocytes. Scale bar in A, D, F, I, and K: 500 µm. Scale bar in D´, K´, B, E, G, J, C and H: 100 µm. Scale bar in A´, F´, I´, L, G and J´: 20 µm.

After providing a macroscopic description, we used Masson’s trichrome staining to characterize pre-amputation tissues at 0 days post-amputation (dpa) and regenerating tissues at 1 dpa, 18 dpa, 28 dpa, 42 dpa, and 180 dpa, as observed in transversal sections *(n = 3 – n = 6 animals for each time*)(Fig 2A–2J and 3A–3L–3L). In the pre-amputated tissue, we can see the different layers of the skin, including the epidermis, dermis, and hypodermis (Fig 2A). The epidermis consists of stratified epithelial tissue, where Leydig cells with granular cytoplasm are visible; this is characteristic of amphibian skin, as described by Seifert *et al.* [51] (Fig 2A’). Below the epidermis, the dermis contains a thin layer composed of the basal lamina, collagen fibers, and fibroblasts (Fig 2A’). Additionally, we can identify Meckel’s cartilage, a type of hyaline cartilage arranged as bilateral bars that are slightly separated by the mentonian symphysis (Fig 2A and Fig 2B). Adjacent to this, we observe the anterior intermandibular muscle (Aim) and the bilateral dental bone, which features dental germs in various developmental stages (Fig 2A and Fig 2B). Within these, the layers of odontoblasts, dentine, and internal enamel epithelium can be distinguished (Fig 2C).

After amputation, loss of continuity of epithelial and skeletal tissue is evident (Fig 2D‘), as well as remnant muscle tissue adjacent to the bone tissue and the amputation plane (Fig 2E, 2E’). At 1 dpa, it is possible to distinguish a monolayer of epithelial tissue (wound epithelium) continuous with the remnant epithelium covering the entire wound edge (Fig 2F and 2G‘), Meckel’s cartilage, and remnant dental bone bilaterally (Fig 2F’). Some fragmented muscle fibers are observed in the contralateral region adjacent to the remaining skeletal and connective tissue (Fig 2G). At 18 dpa, a thickened regenerated epithelial layer representing the apical epithelial cap (AEC) can be identified, which is continuous with the remaining epithelium (Fig 2H and 2H´). However, basal lamina is absent (Fig 2H’). Of great interest, an accumulation of cells with a fibroblastic appearance (Fig 2H, 2I and 2I´´) is observed in the neoformed mesenchymal tissue, which is located underlying the thickened epithelial layer and between the edges of the remnant skeleton, giving an appearance typical of the histological conformation of an early blastema (Fig 2H, 2I and 2I´´). In addition, an accumulation of extracellular matrix rich in collagen fibers is mainly concentrated at the edge of the skeletal tissue (cartilage)(Fig 2I and 2J). Of great relevance, it was possible to distinguish the presence of fragmenting muscle fibers that give rise to the formation of mononucleated cells (presumed myoblasts) (Fig 2I’) suggesting a possible involvement of muscle-derived mononucleated cells during early blastema formation (Fig 2I and 2I’). Similarly, the cells detached from the periosteum of the remaining bone tissue in histolysis were observed in the vicinity of the forming blastema” (Fig 2J and 2J’).

By 28 dpa, the regenerated epithelial layer exhibits a greater degree of stratification, consisting of 4–5 layers, along with the presence of Leydig cells; however, the basal lamina remains undetectable (Fig 3A and 3A´). In terms of the developing blastema, a more dorsal plane reveals a high density of undifferentiated blastema cells, accompanied by a noticeable increase in matrix production, although there is no clear formation of mature bone tissue at its expected site (Fig 3A and 3B). Additionally, the remnants of the dental bone show signs of active histolysis (Fig 3C). In contrast, in a more ventral amputation plane, cartilage tissue neoformation in the blastema area is evident adjacent to the remnant Meckel’s cartilage (Fig 3D and 3D’). Furthermore, melanophore-like pigment cells can be seen in the distal region of the blastema (Fig 3E and 3E’). At 42 dpa, the blastema area between the remaining dental bones begins to narrow, and the regenerated epithelial tissue exhibits a highly stratified structure, consisting of 5–6 layers with an evident basal lamina (Fig 3F and 3F’). Near the remaining ends of the dental bones, both regenerated bone and dental tissues are visible (see Fig 3G). Additionally, in a more ventral section, the regeneration of intermandibular muscle fibers is observable (Fig 3H). By 180 dpa, it is possible to observe the regeneration of all tissues constituting the mandible (Fig 3I-3L). The stratified epidermis, complete with Leydig cells and a basal lamina, is visible (Fig 3I’). Additionally, regenerated muscle fibers can be seen inserted into the perichondrium (*pc*) of the newly formed cartilage (Fig 3J and 3J’). In a more dorsal and superficial plane, regenerated dental bone and functional teeth can be observed bilaterally surrounding Meckel’s cartilage, which displays thickening and fusion at the mandibular symphysis region (Fig 3K-3L).

### Regeneration and integration of skeletal tissues after complete transverse amputation of both hemimandibulae

**Fig 4:**
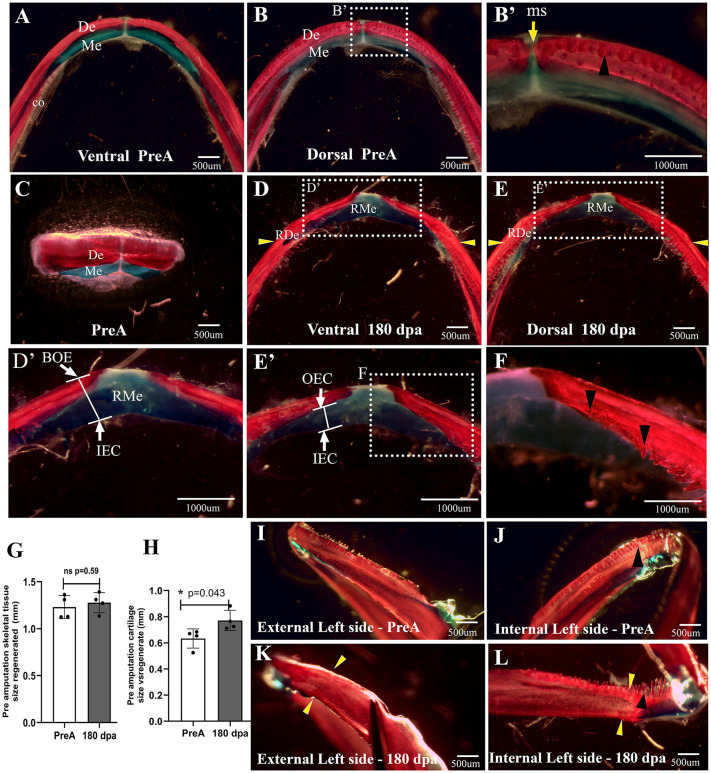
Pre- and post-amputation tissue diaphanization. **(A-B)**, Pre-amputation ventral and dorsal views. (**B**’), Zoom of dental structures and mentonian symphysis (yellow arrow). **(C)**, Frontal view of mandibular tissue removed from the same individual described at 180 dpa in **D. (D)**, Ventral view of regenerated mandible at 180 dpa. (**D’**), Zoom of ventral view of mentonian symphysis. **(E)**, Dorsal view of regenerated mandible at 180 dpa. (**E’**), Zoom of dorsal view of mentonian symphysis. **(F)**, Regenerated teeth attached to the regenerated dental bone. **(G)**, Quantification of the total regenerated skeletal tissue. **(H)**, Quantification of the regenerated cartilage at 180 dpa. (**I**) and **(J)**, external and internal views of the pre-amputation dental bone with full integrity. (**K**) and **(L)**, External and internal views of the regenerated dental bone at 180 dpa. Successful integration of the regenerated bone to the remaining dental bone is observed.. t-student test was used to compare the values of total skeletal tissue and bone against the thickness of pre-amputation skeletal tissues. Data plot mean ± SEM. *p < 0,05. Yellow arrowhead indicates presumed amputation plane. Black arrowhead indicates dental structures. White arrows indicate the bony outer edge (BOE), inner edge of cartilage (IEC) and the outer edge of the cartilage (OEC). **co**, coronoid bone. **De**, dental bone.. **Me**, Meckel’s cartilage. **ms**, mentonian symphysis. **RDe**, Regenerated dental bone. **RMe,** Regenerated Meckel’s cartilage.

In light of the macroscopic observations, diaphanization was conducted to assess the regeneration of skeletal tissues at 180 dpa and to compare these with the pre-amputation tissues (Fig 4A-4J). In the pre-amputation tissues, the presence and continuity of the dentary and coronoid bones surrounding Meckel’s cartilage are evident along its entire length, from the presumed coronoid process to the mandibular symphysis, where there is a lack of complete fusion between the bilateral skeletal components (Fig 4A-4C). Furthermore, functional teeth (those attached to the bones) and non-functional teeth (those separated from the bones) can be observed, arranged in parallel rows and distributed across both bones (Fig 4B and 4B’).

Results indicate that at 180 dpa (*n = 4*), skeletal tissues such as dental bone and Meckel’s cartilage were regenerated (Figs 4D and 4E). However, at the distal point near the midline, both the regenerated Meckel’s cartilage and dental bone display atypical and irregular morphology with an increased volume of Meckel’s cartilage (Figs 4D, 4D‘, 4E, and 4E’), compared to the original amputated tissue (Fig 4C). It can be seen that Meckel’s cartilage is not evenly distributed across the ventral surface and extends distally and dorsally where the dentary bone would be expected to be (Figs 4A, 4D and 4D´). Thus, the mandibular symphysis line is poorly defined or absent (Figs 4D‘and 4E’). The regenerated bone tissue is thinner and does not extend fully towards the midline (Fig 4C-4E’). Additionally, floating and functional teeth were observed along the dental bone (Fig 4F). Subsequently, the total vestibulo-lingual thickness of the regenerated skeletal tissue (comprising dental bone and Meckel’s cartilage) was assessed by measuring the distance from the outer vestibular bony edge of the dental bone to the inner lingual edge of the regenerated Meckel’s cartilage (Fig 4D’). The results, with a *p-value* = 0.59, indicate that there is no statistically significant difference; the mean thickness of the regenerated skeletal tissue at 180 days post-amputation (dpa) was 1.27 mm ± 0.075, compared to a pre-amputation mean of 1.22 mm ± 0.052. This finding suggests that the thickness of the amputated skeletal tissue has been successfully regenerated (Fig 4G). In contrast, when examining the thickness of regenerated versus pre-amputation cartilage (Fig 4E’), with a *p-value = 0.04* a statistically significant difference was observed. The pre-amputation cartilage had a mean thickness of 0.63 mm ± 0.029, while at 180 dpa, it measured 0.7909 mm ± 0.047 (Fig 4H). This supports the previously described qualitative observations of increased Meckel’s cartilage volume (Fig 4D-4E). Additionally, it was found that the mean ± SEM of the thickness of the regenerated bone at 180 dpa was 0.4802 ± 0.08021. Meanwhile, the mean ± SEM of the thickness of the bone prior to amputation was 0.6040 ± 0.04933. These results show that the regenerated bone was thinner than the pre-amputation bone at this point of evaluation, contrary to what occurred in the case of cartilage tissue.

Of great interest, there is a bilateral continuity between the remaining dental bone and the regenerated bone, indicating successful integration and making it difficult to identify the precise location of the amputation plane (Fig 4K and 4L). This suggests an osseous integration reminiscent of the pre-amputation tissue (Fig 4I–4L).

### Regenerating mandibular tissues express genes associated with cellular dedifferentiation and blastema formation after complete transverse mandibular amputation

**Fig 5:**
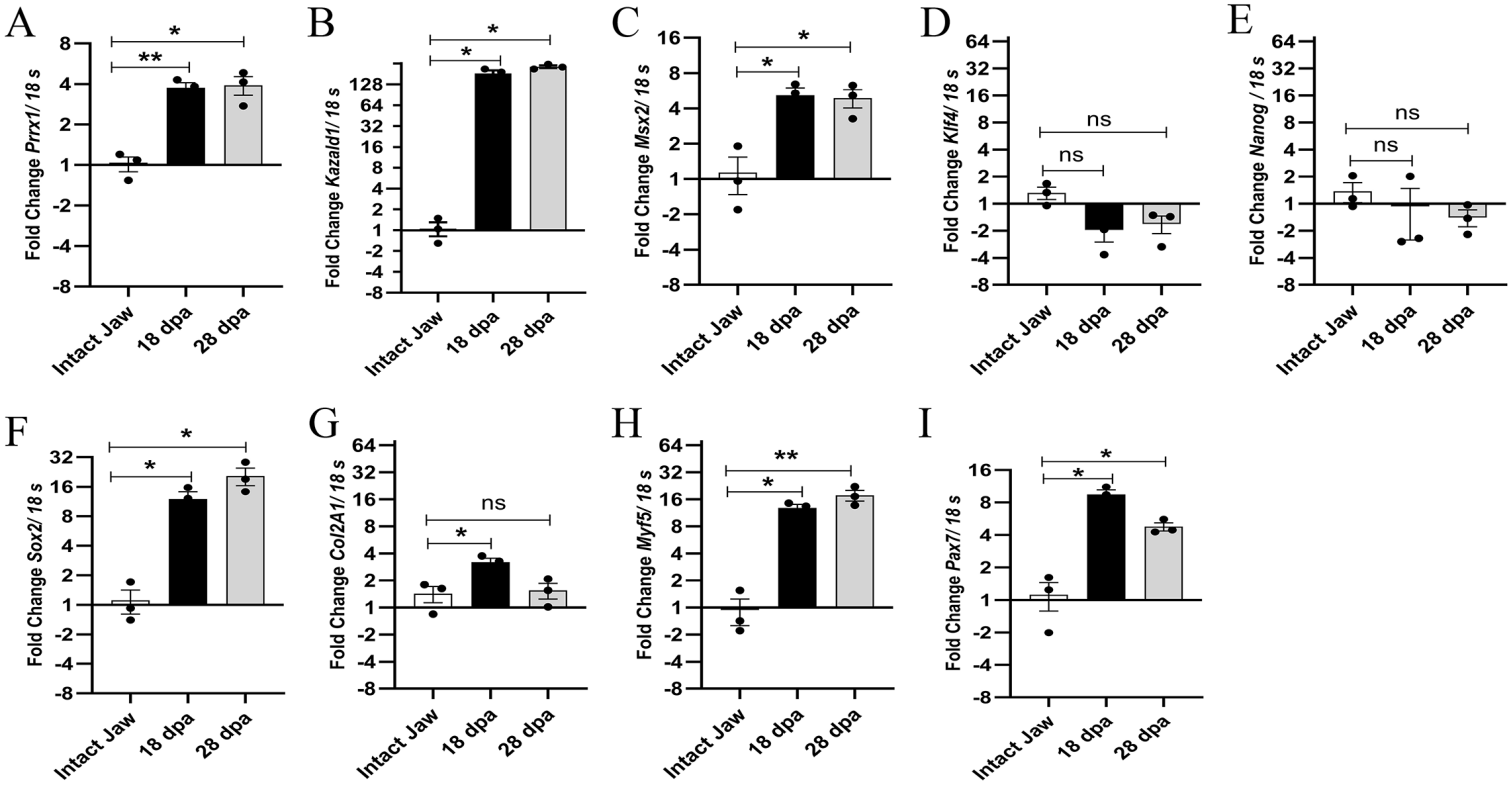
RT-qPCR of genes associated with cell dedifferentiation and blastema formation. (A-I) Expression levels of *Prrx1, Kazald1, Msx2, Klf4, Nanog, Sox2, Col2A1, Myf5* and *Pax7* genes at 18 dpa and 28 dpa compared to basal levels of intact mandible. Data were normalized according to the reference gene 18S. Unpaired independent samples t-student test was performed. Data plot mean ± SEM. *p < 0.05, **p < 0.005, ns: not significant.

Considering the morphological and histological descriptions of the blastema formed after complete transverse mandibular amputation, we adopted a targeted, candidate-gene approach to evaluate the activation of cellular programs associated with regeneration. Gene selection was based on a comprehensive literature review in *Ambystoma mexicanum*, focusing on genes repeatedly implicated in cellular dedifferentiation, plasticity, and blastema formation. Specifically, we analyzed the expression of genes associated with mesenchymal dedifferentiation (*Prrx1, Msx2*), early blastema signaling (*Kazald1*), cellular plasticity and stemness (*Klf4, Nanog, Sox2*), chondrocyte dedifferentiation (*Col2a1*), and myogenic lineage activation (*Pax7, Myf5*) [45,47,48,52–57].To evaluate the possible dedifferentiation of fibroblasts and their contribution to blastema formation, we assessed the expression of the *Prrx1* gene, observing a significant increase in expression levels at 18 dpa and 28 dpa compared to basal levels in the intact mandible (Fig 5A). Of great interest, we identified an overexpression of the *Kazald1* gene at 18 dpa and 28 dpa compared to intact jaw levels, which has been suggested to be a specific gene for epimorphic regeneration in limbs (Fig 5B) [48]. Additionally, at 18 dpa and 28 dpa, we detected significant expression of the *Msx2* gene, which has been implicated in the early stages of regeneration and blastema formation (Fig 5C). Likewise, we evaluated the expression of genes associated with cellular reprogramming, such as *Klf4, Nanog* and *Sox2*, of which *Sox2* evidenced significant levels of expression for 18 dpa and 28 dpa. At the same time, *Klf4* and *Nanog* did not show significant differential expression levels for the days evaluated, compared to basal levels in the intact mandible (Fig 5D-5F).

On the other hand, the expression level of the *Col2a1* gene involved in chondrocyte dedifferentiation [54,55], was evaluated, finding significant levels at 18 dpa, while at 28 dpa no differential expression was found compared to basal levels in the intact mandible (Fig 5G). Finally, considering the presence of muscle tissue in the amputation plane and its potential contribution to blastema formation, the expression of muscle precursor marker genes such as *Myf5* and *Pax7* was evaluated [57]. The data show that *Myf5* and *Pax7* were overexpressed at 18 dpa and 28 dpa compared to pre-amputation basal levels (Fig 5H-5I).

### Characterization of cell proliferation after mandibular transverse amputation in the *A. mexicanum* model

**Fig 6:**
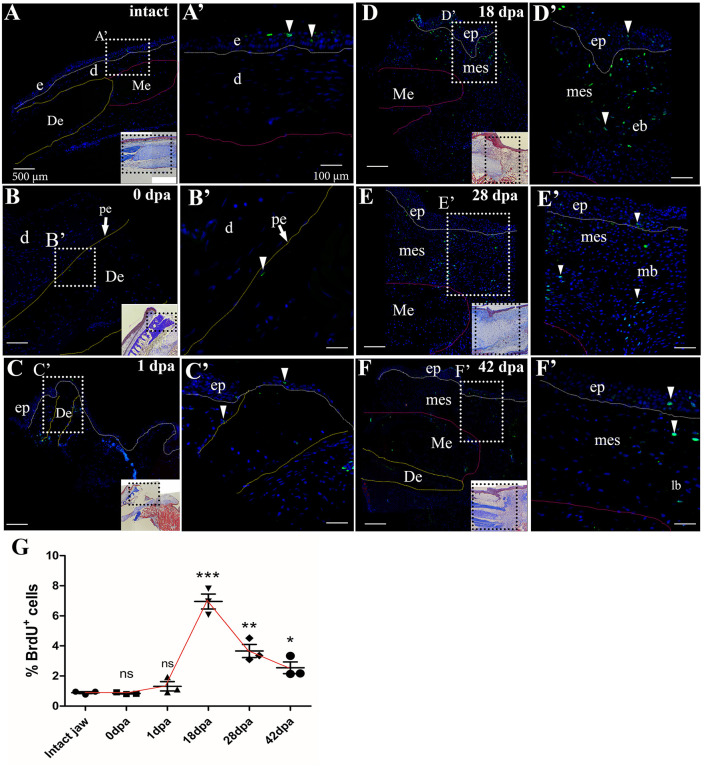
Quantification of post-amputation mandibular cell proliferation by immunofluorescence against BrdU. **(A)**, BrdU^+^ cells in intact mandible. (**A’**), Magnification of A, epidermis and dermis area. **(B)**, Cell proliferation at 0 dpa, BrdU-positive cell located at the periosteal border of the remaining bone tissue. (**B’**), Magnification of **B. (C)**, Cell proliferation at 1 dpa. BrdU^+^ labeling is observed at the edge of the remnant bone tissue and epithelium lining the wound edge. (**C’**), Magnification of **C. (D)**, BrdU-positive cells at 18 dpa. (**D’**), Enlargement of **D. (E)**, BrdU^+^ cells at 28 dpa. (**E’**), Magnification of **E. (F)**, BrdU^+^ cells at 42 dpa located in the epithelial zone and regenerating mesenchyme. (**F’**), Magnification of **F. (G)**, Quantification of the percentage of BrdU^+^ cells for intact mandible at 0 dpa, 1 dpa, 18 dpa, 28 dpa, and 42 dpa. One-way ANOVA with Tukey’s post hoc test for multiple comparisons was used to compare baseline levels of proliferation versus post-amputation days evaluated. Data plot mean ± SEM. *p < 0.05, **p < 0.01, ***p < 0.001, ns: not significant. Dotted white lines delimit epithelium and bone tissue, white arrowhead indicates BrdU^+^ cells, **e**: epidermis; **d**: dermis; **mes**: mesenchyme. The histologic images are a guide to the area of the immunofluorescence sections described. Scale bar in A-E: 500µm. Scale bar in insets (A´-E´): 100 µm.

To identify and characterize proliferation events during mandibular regeneration, we assessed this event using 5-Bromo-2-deoxyuridine (BrdU). Low levels of proliferation were detected in the intact mandible, with an average of 0.9% BrdU^+^ cells observed mainly in the epidermis (Fig 6A, 6A’). After immediate mandibular amputation (0 dpa), proliferation levels were similar to baseline levels with no significant differences (*p > 0.05*), ranging from 0.79% to 0.88% (Fig 6B, 6b´, and 6G). In this way, proliferating cells were labeled to the periosteum of the remaining bone tissue (Fig 6B, 6B’). At 1 dpa, proliferation levels increased slightly with BrdU^+^ cells located around the amputation plane and remnant bone tissue (Fig 6C, 6C’); however, these proliferation levels were not statistically different from the baseline (Fig 6G). Of great relevance, at 18 dpa, the percentage of proliferating cells increased to 7.5%, reaching the peak of maximum proliferation, significantly different from those detected in the intact mandible (*p < 0.001*) (Fig 6G). At this stage, the proliferating cells were concentrated in the forming blastema, the regenerating stratified epithelium, and the mesenchyme surrounding the remaining bone tissue. (Fig 6D, 6D’).

At 28 dpa and 42 dpa, the proliferation levels decreased considerably (3.6% and 2%, respectively) compared to those found at 18 dpa (Fig 6G). BrdU^+^ cells were detected mainly in the mesenchyme adjacent to the regenerating epithelium, as well as in epithelial cells. (Fig 6E, 6E’-6F’). These levels of proliferation remained statistically different from those detected in the intact mandible, with *p < 0.05* and *p < 0.01* for 28 dpa and 42 dpa, respectively. These results suggest that while there is a significant increase in proliferation levels at 18 dpa, 28 dpa, and 42 dpa, these are relatively low, which could correlate with the slow growth of regenerating tissues described above.

### Second proximal mandibular transverse amputation promotes a failed regenerative response

**Fig 7:**
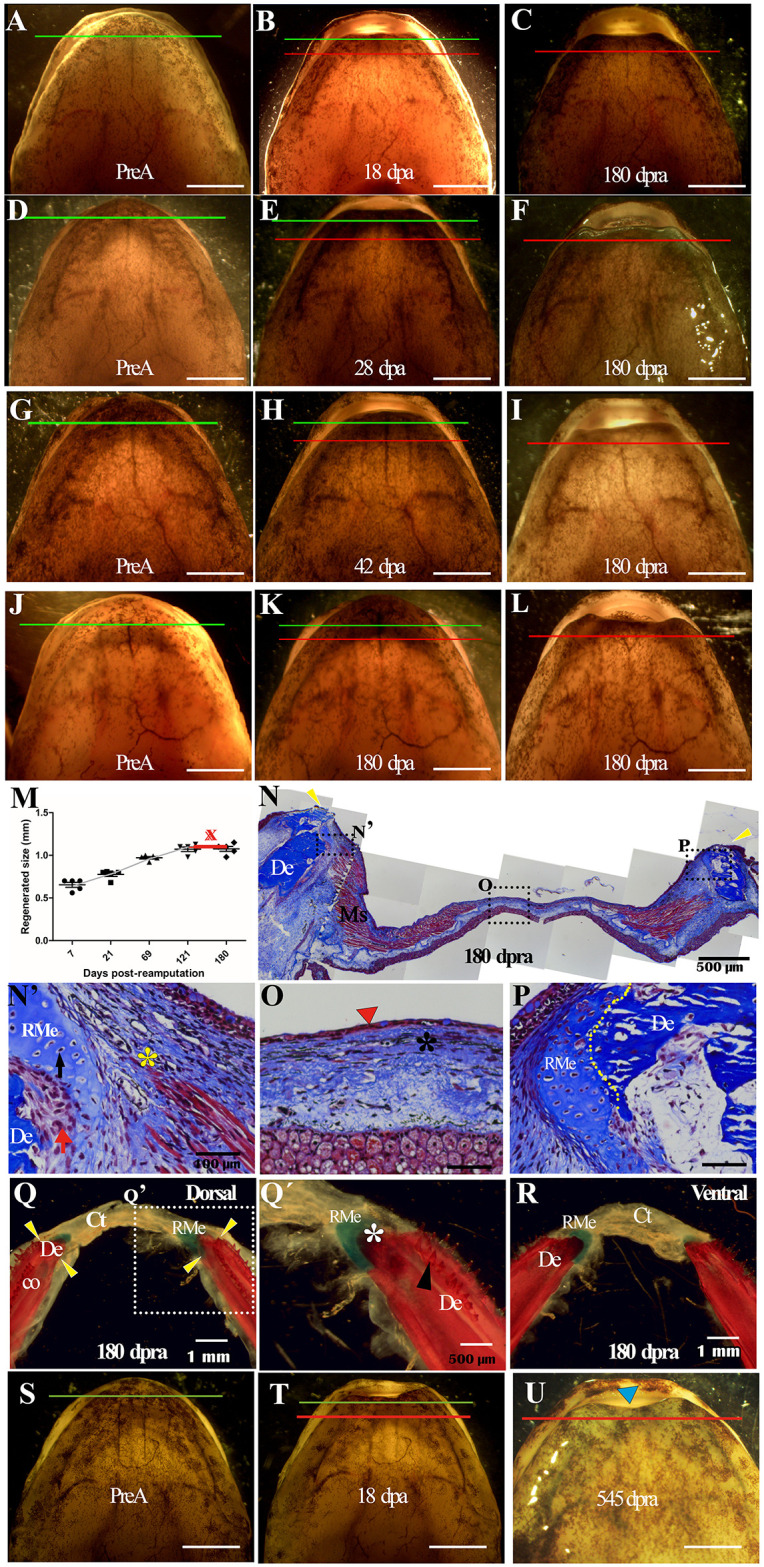
Regenerative response post second transverse mandibular amputation in a more proximal plane. (**A, D, G, J)** In the pre-amputation tissue, the green line indicates the first amputation plane (1st ap). (**B, E, H, K**) Regenerating tissue after the first amputation. The red line indicates the second amputation-cutting plane. (**C, F, I, L**), regenerated tissue at 180 dpr; scale bar = 5 mm. **(M)** Quantification of regenerated tissue after second amputation at 7 dpr, 21 dpr, 69 dpr, 121 dpr, and 180 dpr. **(N)** Representative histologic section at 180 dpr. Remnant bilateral skeletal and muscle tissue as well as regenerated epithelial and connective tissue overlying the remnant tissue. (**N’**) Magnification of the muscle tissue insertion zone to the little regenerated perichondrium (yellow asterisk), black arrow indicates chondrocytes; red arrow (accumulation of mesenchymal cells). **(O)** Magnification of epithelium (red arrowhead) and fibrotic connective tissue (black asterisk) regenerated in central and medial zone. **(P)**, Magnification of the contralateral skeletal tissue zone. The yellow line indicates the boundary between remnant bone and regenerated cartilage. **(Q)** Diaphysis of the mandible at 180 dpr. Dorsal view shows bilateral dentary bones and Meckel’s cartilage, joined by regenerated connective tissue (Tc). Yellow arrowheads indicate presumed amputation plane. (**Q’**) Enlargement of Meckel’s cartilage continuity zone with regenerated dental bone (white asterisk). Dental structures are seen in remnant dentary bone (black arrowhead). **(R)** Ventral view of mandible at 180 dpr. **(S-U)**, Representative animal after a second proximal amputation (reamputation) at 545 days post-reamputation. Scale bar A-L; S-U = 5 mm. and N´-P = 100 um;.

Given the success of the regenerative response seen after transverse amputation of the entire mandible, we aimed to evaluate the regenerative response when a second, more proximal amputation was performed. For this purpose, we followed up for 180 days post-reamputation (dpr) on those animals that had tissues harvested at 18 dpa, 28 dpa, 42 dpa and 180 dpa (*n = 3* animals for each time) (Fig 7A-7L). After the second amputation, although regenerative tissue was seen, it collapsed, resulting in regenerative failure both morphologically and structurally, unlike what was observed after the first distal amputation (Fig 7A-7L). The quantification of regenerating tissue after the second amputation showed that at 180 dpr, only 1 mm of the amputated tissue had regenerated. Additionally, the growth of regenerating tissue was quite slow and ceased at 121 dpr (Fig 7M). Significantly, subsequent evaluations of animals performed 545 days (approximately 1.5 years) after the second proximal amputation (reamputation) show that during this period, the amputated tissue was not regenerated (Fig 7S-7U). In these animals, the external anatomy of the amputated tissue is not restored (Fig 7U). These findings suggest that the most proximal tissues of the mandible in this species undergoing reamputation exhibit a decline in their regenerative response.

At 180 dpr *(n = 3*), histological analysis shows that in addition to the remaining skeletal tissues (dental bone and Meckel’s cartilage) and remaining muscle tissue, only dense regenerated connective tissue and regenerated epithelium covering the remaining tissue can be identified along the entire length of the amputation plane (Fig 7N). Furthermore, there is no proximal-distal continuity of regenerated skeletal tissue between the two hemimandibles, and regenerated dental and muscular tissue is absent (Fig 7N). On the other hand, adjacent to the amputation plane, there is an accumulation of mesenchymal cells between the edge of the remnant dental bone and the remnant cartilage, as well as muscle fibers inserted in the perichondrium of the remnant skeletal tissue (Fig 7N’).

Of great interest, at the level of the central (medial) zone of the regenerated tissue, the regenerated epithelium is thin and has only 1–2 layers of cells without their basal lamina (Fig 7O); likewise, underlying this epithelium, a dense connective tissue with a compact extracellular matrix rich in collagen fibers with a healing aspect can be distinguished; however, towards the more proximal region, this matrix is less compact (Fig 7O). On the other hand, in the contralateral region of the amputation plane, over the remaining dental bone, a slight continuity of apparently regenerated Meckel’s cartilage is observed (Fig 7P). To provide a comprehensive and macroscopic description of the regenerated skeletal tissue, the diaphanization technique was performed (Fig 7Q and 7R). The results show that adjacent to the amputation plane, a small bone fragment representing a portion of the regenerated dental bone can be distinguished on which a small portion of cartilaginous tissue continues, compatible with a portion of the regenerated Meckel’s cartilage (Fig 7Q and 7Q’). Consistent with the histological data, the ends of the hemimandibles are joined together by regenerated fibrous connective tissue where the regenerated skeletal tissue was expected to take place (Fig 7Q and 7R).

**Fig 8:**
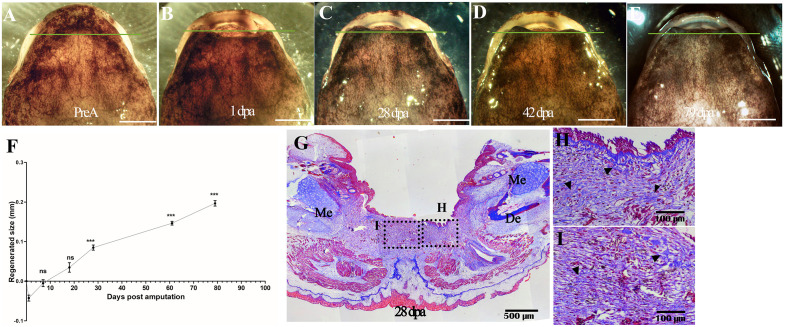
Regenerative response following proximal amputation in animals without prior intervention. Regenerative response following proximal amputation in animals without prior intervention. **(A-E)**, representative images of regenerative response follow-up: pre-amputation, 1 dpa, 28 dpa, 42 dpa, and 79 dpa, respectively. Solid green line indicates proximal amputation plane. **(F)**, Regenerative tissue growth curve. **(G)**, Representative histologic section at 28 dpa. (H-I), Magnification of regenerated tissue showing the presence of dense connective tissue. Black arrowheads: sites of high collagen deposition. Data plot mean ± SEM. *p < 0.05; ** p < 0.01; *** p < 0.001. **PreA**: Pre-amputation, dpa: days post-amputation. **De**, dental bone. **Me**, Meckel’s cartilage. Scale bar A-E: 5 mm.

To assess whether the failure of regeneration was associated solely with reamputation (second regeneration) or with the more proximal position of the amputation plane, a proximal amputation equidistant from the plane of the second amputation (reamputation) was performed in animals that had not previously undergone surgery. Macroscopic analyses show no blastema formation at 28 and 42 dpa. Even at 79 dpa, no blastema formation was observed, and the regenerative response remains collapsed at early stages (Fig 8A-8E). At this point of observation, only 0.2 mm of newly formed tissue was evident (Fig 8F). Histology at 28 dpa shows that between the epithelium and the amputation plane, dense connective tissue rich in collagen fibers (similar to scar tissue) extends beyond the basal lamina, with moderate cell accumulation (Fig 8G-8I).

## Discussion

**Fig 9:**
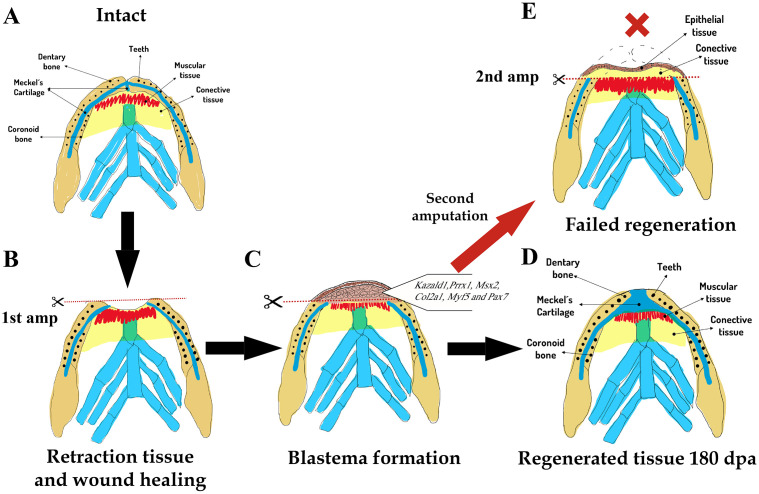
Model of tissue response after complete transverse amputation of the mandible in axolotls. **(A-D)**, Regenerative response after first amputation. **(E)**, Failed regenerative response after second more proximal amputation. All illustrations were created by the authors based on original photographs taken by the authors. The same mandibular illustration shown in Figure 1A is reused here for comparative purposes. The photographs were used exclusively as anatomical references and are not reproduced in the manuscript. No third-party copyrighted material was used. All elements comply with the Creative Commons Attribution (CC BY 4.0) license.

The search for improvements and novel therapeutic strategies in the field of craniofacial tissue regeneration, particularly concerning mandibular tissues, has prompted investigations into the tissue responses and cellular mechanisms that govern regeneration in species capable of regenerating complex structures such as the mandible [5,7,31,32,34–36,58]. The current study assessed the regenerative response of mandibular tissues following complete transverse amputation involving both hemimandibles in adult *A. mexicanum* specimens. Our findings, derived from a macroscopic, histological, and gene expression perspective, demonstrated an epimorphic regenerative response in the amputated mandibles. However, this regenerative response is compromised when a second amputation (reamputation) or single proximal amputation is performed at a more proximal position along the proximal–distal mandibular axis in the animals studied (Fig 9).

### Complete transverse amputation of the mandible in adult *A. mexicanum* promotes a tissue response like that described in other species

Our observations show that after complete transverse amputation of the mandible in adult *A. mexicanum*, there was immediate retraction of soft tissue and complete wound closure at 1 dpa when covered by a single epithelial layer (Fig 1D and 2F-2G). These results are consistent with previous studies on mandibular regeneration in newts [31,32] and zebrafish [34,36]. Additionally, a rapid inflammatory response, cessation of bleeding, and epithelial layer formation were similarly reported after lateral jaw cuts in *A. mexicanum* [7]. Our findings reveal that the muscle at the amputation site showed significant fragmentation and degeneration by 18 dpa, with visible changes from distal to proximal. This was accompanied by remodeling or histolysis of the remaining cartilage and bone. Similar results were observed in *Triturus viridescens*, where muscle fiber degeneration extended proximally toward the base of the mandible during the first week post-amputation, while the dental bone exhibited minimal remodeling [31]. In *Danio rerio,* transverse mandibular post-amputation, fragmented muscle cells were found between the regenerated dermis and remaining muscle after re-epithelialization [35,36]. Additionally, in newt limbs (*N. viridescens*), muscle fragmentation was noted at 14 dpa [57], along with histolysis of skeletal tissues [38,59].

Regarding the time required for amputated craniofacial structures regeneration, it has been noted that this process takes longer than limb regeneration [7,31,32]. In the current study, we found that at 180 dpa, the regenerated mandible was comparable in size, external morphology and histologic characteristics to the original, indicating an advanced stage of structural regeneration. Consistent with our findings, previous research on newts has documented a regeneration period of 180 dpa for *Cynops pyrrhogaster* and 140 dpa for *Triturus viridescens* following complete cross-sections of the mandible [31–33]. Furthermore, studies on lateral cuts of the mandible in adult *Notophthalmus viridescens* indicate that a period of 12 weeks (90 dpa) is required for the complete regeneration of the defect [30]. Of great interest, a recent study in *A. mexicanum* showed that although it was observed that at 90 dpa, mandibular tissues are regenerated, they were 9% smaller than the original tissue, suggesting that more time is required to complete the regeneration process [7]. In line with the above, our study indicated that, despite the amputation plane being completely transverse, 180 days were necessary to achieve near-complete structural restoration of the mandible in the same species. Research conducted on other species, such as *Danio rerio*, has demonstrated that 90 days are required for complete regeneration of the mandible after transverse cuts, while 120 days are needed for lateral sections [34,36,58]. However, the almost linear increase in tissue length observed up to 180 dpa in our study suggests that growth and remodeling may still be ongoing. Therefore, this stage likely represents an advanced phase of structural regeneration rather than a fully stabilized homeostatic state. Thus, future long-term analyses examining tissue stabilization, functional recovery, and the dynamics of progenitor cell populations will be required to determine when mandibular regeneration is fully complete.

From a physiological perspective, our observations indicate that there are no noticeable impairments in the behavior of the animals nor any limitations that hinder their ability to feed properly, despite the invasiveness of the amputation method employed. This implies that the model of complete transverse amputation serves as a viable strategy for studying the underlying mechanisms of the regenerative response described here, while also allowing for comparisons with similar or other types of amputations performed on mandibles of the same or different species. Additionally, it enables comparisons with other structures that undergo regeneration, such as limbs.

### Complete transverse amputation of the mandible in *A. mexicanum* promotes an epimorphic regenerative response and the expression of genes related to this type of regeneration

Epimorphic regeneration has been extensively documented in various structures of animals with regenerative abilities, such as the caudal fin in zebrafish [60,61], as well as the tail and limbs in newts and urodele amphibians like *Ambystoma mexicanum* [1,40,62–64] and, to a lesser extent, in complex structures such as the mandible of these species [7,31,32,34–36].

The present study revealed that following complete transverse amputation of this structure, tissue regeneration progresses through a series of stages similar to those observed during epimorphic regeneration of limbs and jaws in other species [38,59,61,65]. Histological analysis revealed a significant accumulation of mesenchymal cells with a fibroblastoid appearance, likely dedifferentiated cells between the remaining skeletal tissue and the underlying wound epithelium. Similarly, in studies conducted on newts and zebrafish, it has been observed that at three weeks and two days post-amputation of the mandible, respectively, a blastema forms from a mass of cells seemingly derived from the nuclei of degenerated muscle fibers and fibroblasts, which accumulate in the distal regions of the mandibular amputation plane [31,32,35,36]. Furthermore, in lateral mandibular sections of *A. mexicanum*, the emergence of a cluster of mesenchymal cells resembling the blastema noted in limb regeneration has been documented [7].

On the other hand, at 18 dpa, we observed elevated levels of proliferation within both the regenerating epithelium and the blastema mesenchyme. This finding aligns with previous studies on lateral resection of the mandible in *A. mexicanum*, which reported a significant increase in proliferation levels at 14 dpa, specifically within the blastema mesenchyme [7]. Similarly, following a transversal amputation of the mandible in *Cynops pyrrhogaster* newts, high levels of proliferation have been reported at 14 dpa, especially at the mesenchyme level, whose authors conclude that these dedifferentiated cells re-enter the cell cycle, contributing to blastema formation [33].

To validate blastema formation, we assessed markers associated with epimorphic regeneration and specific cell lineage precursors previously identified in limb regeneration [45,47,48,66]. Our findings showed high expression of the blastema marker *Kazald1* and connective tissue dedifferentiation genes *Prrx1* and *Msx2* at 18 dpa and 28 dpa during early and mid-blastema formation (Figure 6A-6C). These results are similar to previous studies in *A. mexicanum*, highlighting *Kazald1* as a marker for epimorphic regeneration due to its high expression in regenerating tissues and absence in non-amputated or developmental tissues [48]. Similarly, *Kazald2* expression has been identified in mesenchymal cells of the blastema following lateral cut amputation of *A. mexicanum* mandibles [7]. Moreover, *Prrx1* and *Msx2* are expressed in dedifferentiated fibroblasts and epidermal cells during limb regeneration [45,56,67]. Of great relevance, post-lateral mandibular amputation in axolotls, an increase in PRRX1 protein expression has been described in proliferating cells of tissues underlying the amputation plane in both early stages and in mesenchymal cells of the mandibular blastema at 14 dpa and 35 dpa [7]. These findings, although derived from a different type of injury than that in our study, are similar to our expression data, where *Prrx1* expression increased significantly at 18 and 28 dpa in tissues derived from the early and middle blastema, respectively. Our findings, alongside these reports, suggest that after complete transverse mandibular amputation, a blastema forms, potentially involving fibroblasts from surrounding connective tissue.

The role of muscle tissue in blastema formation, either through the dedifferentiation of muscle fibers or contributions from satellite cells (Pax7^+^), has been explored [57,68]. Our results show notable expression of *Pax7* and *Myf5* at 18 dpa and 28 dpa in blastema-derived tissues, suggesting the activation of myogenic gene expression programs during blastema formation and muscle regeneration in the mandible of *A. mexicanum*. While qPCR does not provide cellular localization, this transcriptional profile is comparable to previous studies indicating that Pax7^+^ cells can generate proliferating myogenic precursors that aid in blastema and muscle regeneration, even in the absence of muscle fibre dedifferentiation [57]. However, although during limb regeneration in newts, muscle fibre dedifferentiation is the predominant mechanism for blastema formation, reports in *Cynops pyrrhogaster* (newt) show that as in *A. mexicanum* limbs, post-transverse jaw amputation, proliferating Pax7^+^ cells are required for blastema and regenerated muscle formation [33]. Likewise, the expression of *Myf5* and *Pax7* in satellite cells represents the largest population of myogenic precursors that make up the blastema in *A. mexicanum* limbs [57]. Therefore, it is plausible that during mandibular regeneration in *A. mexicanum*, muscle tissue could contribute satellite cells expressing *Pax7* for blastema formation as occurs in limb regeneration in this species and during jaw regeneration in *Cynops pyrrhogaster*. Nonetheless, the possibility of muscle fiber dedifferentiation or a combination of both mechanisms remains to be explored in further studies. Thus, further lineage-tracing approaches will be required to determine the final fate and functional contribution of muscle-derived cells during blastema formation.

In our evaluation of genes involved in cell reprogramming, we found that *Sox2* was significantly expressed during blastema formation at 18 and 28 dpa. This is consistent with previous studies showing notable *Sox2* expression during limb and tail blastema formation in newts and *A. mexicanum* [46,47,62,69]. Additionally, loss of *Sox2* function reduces neural precursor proliferation during tail regeneration in *A. mexicanum* [46], suggesting a role in the dedifferentiation and proliferation of blastema-forming cells in axolotl mandibular regeneration. Conversely, *Klf4* and *Nanog* did not show significant expression changes, indicating they may not have a function at the assessed time points. Further investigation of earlier stages of mandibular regeneration is needed to clarify the roles of these genes.

Finally, previous studies of lateral mandibular injury in axolotl have shown that regeneration proceeds through the formation of two spatially distinct blastemas within a single hemimandible, one arising from the distal remnant and another from the proximal remnant [7]. Notably, these blastemas exhibit asynchronous differentiation, with the distal blastema differentiating first and extending cartilage toward the proximal stump [7]. In contrast, complete transverse mandibular amputation in our model leads to the formation of a single, continuous blastema spanning the entire amputation plane. From this unified blastema, bilateral hemimandibular structures regenerate simultaneously and subsequently fuse at the midline, restoring mandibular continuity. This mode of regeneration differs fundamentally in blastema organization and spatial patterning from lateral injury models, despite sharing core epimorphic features such as blastema formation and progenitor-associated gene expression. Importantly, a similar single-blastema response has been reported following complete transverse mandibular amputation in other salamander species, including newts [30,31], suggesting that this regenerative architecture represents a conserved response to complete mandibular transection in urodelan amphibians. However, further studies are needed to thoroughly elucidate the cellular and molecular mechanisms, as well as the contribution of the remaining tissues to the formation of the blastema and its derivatives after a complete transverse injury, and whether these differ from those that regulate the regenerative response in lateral jaw amputations in axolotls.

### Complete transverse amputation promotes integration of regenerated skeletal tissues, but is not morphologically like pre-amputation tissue

Post-complete transverse amputation of the mandible in *A. mexicanum*, skeletal tissues such as Meckel’s cartilage and dentary bone regenerate bilaterally at 180 dpa and fully integrate with the remaining tissue; however, the regenerating dentary bone does not entirely cover the regenerated cartilage along its proximal-distal extent. Additionally, in the mandibular symphysis region, an atypical fusion of bilateral Meckel’s cartilages with overgrowth was observed compared to the intact mandible (figure 4A-4B and 4D-4D). Similar results during mandibular regeneration in newts [31,32] describe that the regenerated dentary bone envelops the ventral and lateral sides but not the lingual side of the regenerated cartilage and is separated by a cartilaginous median symphysis for at least six months [32]. On the other hand, in *A. mexicanum*, it has been reported that after lateral mandibular resection, although the regenerated tissue was integrated with the remaining structures, the regenerated cartilage was thicker than the original Meckel’s cartilage, as well as the dentary bone does not entirely cover the regenerated cartilage [7].

On the other hand, in zebrafish, after transverse amputation in the distal third of the mandible, differences have been found in the regenerated mandibular tissues compared to the original [34,36,58]. Thus, at 60 dpa, Meckel’s cartilage was not maintained, and the mandibular bones on each side were joined by a bony matrix at the mandibular symphysis [34,36], evidencing morphologically incomplete regeneration. Our results and previous findings suggest that in regenerating vertebrates, although regenerated mandibular tissues integrate with remnant tissue, regeneration tends to be morphologically incomplete. Accordingly, our observations suggest that the transverse amputation model described in this study represents an opportunity to study the mechanisms that regulate the proper integration of regenerated bone tissue into remnant bone tissue.

### The adult *A. mexicanum* mandible has a limit of regenerative response that depends on the location of the amputation plane in the proximal-distal axis

*A.mexicanum* is known for its remarkable regenerative abilities across various structures throughout its life, including its tail and limbs. To date, no limitations on its regenerative responses have been identified concerning the amputation plane along the proximal-distal axis [12,65,70]. However, in the present study, we demonstrated that re-amputation of the regenerated mandible in a plane more proximal to the initial amputation and single proximal amputation resulted in a collapse of the regenerative response, suggesting a limit to this regenerative process (Fig 7A-7M and Fig 8A- 8I). Thus, by 180 dpr only a thin layer of epithelium and regenerated compact fibrous connective tissue was distinguishable, and even 545 days after reamputation, mandibular regeneration remained collapsed (Fig 7N-7P and Fig 7S-7U). On the other hand, preliminary observations up to 79 days after single proximal amputation also show a reduction in the regenerative response (Fig 8A-8I). Similar results were reported in studies in *N. viridescens* [30,31], where post proximal amputation, regenerated mandibular tissue fails to regenerate half of the amputated mandible, and this tissue is characterized by a defective morphology compared to the morphology of regenerated mandibles when the amputation plane was performed more distally [31]. Although regeneration after a single proximal amputation in naïve animals has not progressed beyond early stages up to 79 days post-amputation, longer-term analyses will be required to determine whether regenerative failure is permanent. Nevertheless, these observations suggest that reduced regenerative capacity following proximal amputation is not exclusively dependent on prior regenerative events.

In addition, and similar to what was found in the present study, authors describe that post mandible proximal amputation in *N. viridescens* the remaining tissues were covered by a thinly thickened epithelial tissue and connective tissue scar [30]. Consistent with the above, it has been reported that repeated amputations at the same amputation plane in *A. mexicanum* limbs limit the regenerative capacity of this structure, resulting in less stratified epithelial tissue and the presence of fibrotic tissue with extensive collagen deposition in the dermis (scar tissue) [71]. Therefore, our results suggest that this regenerative failure may be associated with the formation of scar tissue that overwhelms the regenerative response.

Moreover, limb regeneration failure in *A.talpoideum* and *A.punctatum* is associated with the absence of the apical epithelial cap conformation [72,73], which is known to be a signaling center necessary for blastema formation [37,59,74]. Considering these reports, it is further hypothesized that the lack of regenerative response described in our study may be associated with a failure to form the apical epithelial layer, leading to an absence of the blastema and, ultimately, to failed regeneration of the amputated mandibular structures.

## Supporting information

S1 Dataset Raw data for growth curve, size, and regenerated area analyses. (PDF)

S2 Dataset Raw data for regenerated skeletal tissue size análisis. (PDF)

S3 Dataset Raw data for cell proliferation (BrdU) and regenerated length after proximal mandibular transverse amputation. (PDF)
