# Supplementary material for: Axolotl mandible regeneration following complete transverse amputation involves a blastema formation and exhibits a limit along the proximodistal axis
Source: PLoS One. 2026 May 21;21(5):e0348286. doi: 10.1371/journal.pone.0348286 (PMC13193420; doi:10.1371/journal.pone.0348286)
Supplement: S1 Dataset — (PDF) [file pone.0348286.s001.pdf]

S1 Dataset. Dataset containing raw data from the growth curve analysis, size, and regenerated area deposited in Figshare. **Table S1**. Raw data for the growth curve up to 180 days post-amputation, which were plotted in Figure 1I. **Table S2**. Raw data for length pre-amputation and 180 days post-amputation which were plotted in Figure 1J. **Table S3**. Raw data on pre-amputation area and 180 days post-amputation which were plotted in Figure 1K.

Table S1. Raw data for the growth curve up to 180 days post-amputation.

	Regenerated size (mm)				
dpa	Sample 1	Sample 2	Sample 3	Sample 4	Sample 5
0	0	0	0	0	0
1	-1,11	-0,55	-0,81	-0,75	-0,38
2	-1,05	-0,51	-0,62	-0,63	-0,17
4	-0,87	-0,43	-0,49	-0,5	-0,07
7	-0,57	-0,161	-0,253	-0,4032	0,0722
10	-0,5	-0,0698	-0,196	-0,198	0,1976
14	-0,19	0,067	0,0206	0,0186	0,2888
18	0,04	0,2038	0,089	0,0756	0,2308
21	0,11	0,3292	0,1688	0,1212	0,722
28	0,26	0,4204	0,3968	0,144	0,8588
32	0,68	0,4888	0,4538	0,2352	0,9956
42	1,16	0,7054	0,5336	0,315	1,0754
52	1,55	0,8536	0,6134	0,4404	1,2122
61	1,66	0,922	0,6704	0,6	1,2806
69	2,059	1,0018	0,8072	0,8052	1,3604
80	2,1388	1,15	0,944	0,8736	1,4972
90	2,3326	1,3438	1,3316	1,0104	1,634
101	2,458	1,4236	1,514	1,17	1,7138
111	2,6632	1,5034	1,6736	1,3638	1,7936
121	2,7772	1,6174	1,8104	1,455	1,8506
132	2,9368	1,72	1,8902	1,5234	1,9304
142	3,0964	1,9138	1,9586	1,6602	2,0672
154	3,1648	1,9708	2,1638	1,8768	2,147
164	3,2902	2,1304	2,2208	2,025	2,2838
174	3,427	2,29	2,312	2,3504	2,412
180	3,5638	2,5636	2,4374	2,5302	2,4202

Table S2. Raw data for length pre-amputation and 180 days post-amputation

	Regenerated size (mm)	
	PreA	180dpa
Sample1	2,51	3,5638
Sample2	2,64	2,5636
Sample3	2,64	2,4374
Sample4	2,51	2,5302
Sample5	2,57	2,4202

Table S3. Raw data on pre-amputation area and 180 days post-amputation.

	Regenerated size (mm^2)	
	PreA	180dpa
Sample1	20,3	18,21
Sample2	28,11	25,61
Sample3	26,62	25,89
Sample4	23,73	27,16
Sample5	39,4	37,23
