# Supplementary material for: Axolotl mandible regeneration following complete transverse amputation involves a blastema formation and exhibits a limit along the proximodistal axis
Source: PLoS One. 2026 May 21;21(5):e0348286. doi: 10.1371/journal.pone.0348286 (PMC13193420; doi:10.1371/journal.pone.0348286)
Supplement: S2 Dataset — (PDF) [file pone.0348286.s002.pdf]

S2 Dataset. Dataset containing raw data from the analysis of regenerated skeletal tissue size deposited in Figshare. **Table S1**. Raw data on total regenerated skeletal tissue size at 180 days post-amputation, which was plotted in Figure 4G. **Table S2.** Raw data on the thickness of regenerated cartilage 180 days post-amputation, which was plotted in Figure 4H.

Table S1.Raw data on total regenerated skeletal tissue size at 180 days post-amputation

	**Skeletal tissue size regenerated (mm)**	
	PreA	180dpa
Sample1	1,309	1,391
Sample2	1,128	1,134
Sample3	1,123	1,31
Sample4	1,3614	1,271

Table S2. Raw data on the thickness of regenerated cartilage 180 days post-amputation

	**thickness of regenerated cartilage**	
	PreA	180dpa
Sample1	0,654	0,785
Sample2	0,671	0,7246
Sample3	0,525	0,883
Sample4	0,684	0,712
