# Supplementary material for: Axolotl mandible regeneration following complete transverse amputation involves a blastema formation and exhibits a limit along the proximodistal axis
Source: PLoS One. 2026 May 21;21(5):e0348286. doi: 10.1371/journal.pone.0348286 (PMC13193420; doi:10.1371/journal.pone.0348286)
Supplement: S3 Dataset — (PDF) [file pone.0348286.s003.pdf]

S3 Dataset . Dataset containing raw data on cell proliferation levels expressed as a percentage of BrdU-positive cells and length of the regenerated after proximal mandibular transverse amputation. These data were deposited in Figshare. **Table S1**. Raw data on the percentage of BrdU-positive cells in intact tissue at dpa, 1dpa, 18 dpa, 28 dpa, and 42 dpa. These data were plotted in Figure 6G. **Table S2**, Length of the regenerated section following proximal amputation in animals without prior intervention at 0, 1, 7, 18, 28, 61 and 79 dpa. These data were plotted in Figure 8F.

Table S1. Percentage of BrdU-positive cells after mandibular transverse amputation

	percentage of BrdU-positive cells										
	**Intact jaw (%)**		**0dpa**		**1dpa**		**18dpa**		**28dpa**		**42dpa**
Sample 1	0,95	Sample 1	0,80	Sample 1	0,91	Sample 1	6,96	Sample 1	4,52	Sample 1	2,13
Sample 2	0,95	Sample 2	0,91	Sample 2	1,93	Sample 2	6,08	Sample 2	3,36	Sample 2	3,34
Sample 3	0,77	Sample 3	0,81	Sample 3	1,10	Sample 3	7,81	Sample 3	3,11	Sample 3	2,17

Table S2. Length of regenerated tissue after proximal amputation without prior intervention

	Length of the regenerated section (mm) after proximal amputation			
**dpa**	**Sample 1**	**Sample 2**	**Sample 3**	**Sample 4**
0	0	0	0	0
1	-0,05	-0,03	-0,06	-0,03
7	-0,02	0,01	-0,02	0,01
18	0,02	0,06	0,01	0,05
28	0,08	0,1	0,07	0,09
61	0,14	0,146	0,14	0,16
79	0,195	0,19	0,185	0,217
